# A Letter from Mr. Blair to Dr. Bradley, on Cancerous Hydatids

**Published:** 1801-09

**Authors:** W. Blair

**Affiliations:** Great Russel Street, Bloomsbury


### Medical and Phyfical Journal.

VOL. VI.] SEPTEMBER, 1801. [NO. XXXI.

### *A Letter from Mr.* BLAIR *to Dr.* BRADLEY, *on Cancerous Hydatids.*

DEAR SIR,

IF the following Cafe fhould appear to you fufficiently interefting to lay before the public, and tends in any degree to illuftrate the opinion of Dr. Adams on the nature of cancerous complaints, it is at your entire difpofal. But, in communicating a particular fact, I wifh it to be underftood, that I do not pledge myfelf to fupport a fyftem or a theory: nor do I think this to have been the defign of Dr. Adams, when he publifhed his Obfervations on Morbid Poifons;" for, in the chapter "OF CARCINOMA AND OTHER LOCAL DISEASES USUALLY CALLED CANCER," he exprefsly fays, "I am only propofing enquiries, without venturing to form theories."

So far as I can collect from his work on Morbid Poifons, it appears, that the exiftence of cyfts or hydatids, poffeffed of a life independent of the fubject in which they grow, is regarded by the author as conftituting the effential character of the "true carcinoma." By the term carcinoma he intends to defignate all thofe affections which (properly fpeaking) have been denominated fcirrhus and cancer; and although he "knows of nothing that can difcriminate early carcinoma from other tumours," this difeafe is fuppofed always to affume the animalcular form.

The chief fubject of controverfy, I think, will be refpecting the *genuineness* of the complaint, and the diftinction of *carcinomatous hydatids*. In cancerous cafes, where the hydatid is found, perhaps no queftion will arife; but, where this animal is wanting, may it not be urged that the diforder was not really cancerous? "In all the cancerous breafts, tefticles, and tumours, which I have examined," fays a very recent writer, "I never faw any thing which could be confidered diftinctly as a hydatid." (Burns' Differt. on Inflam. Vol. II.) Until lately I could have faid the fame; but the cafe I have juft met with, will make me more circumfpect in my examination of cancerous fubftances.

On the 15th of May 1801, Mr. Chambers, a furgeon at Deal, fent me a letter by one of the pilots of that town, requefting my opinion on the nature of a fwelled tefticle, under which the bearer had laboured a confiderable time. The tumour fo nearly refembled a true fcirrhus, that Mr. Chambers wifhed the patient to fuffer its extirpation. My opinion entirely coincided with his; and as the fpermatic chord was becoming much enlarged and thickened, my advice was that the teftis fhould be removed immediately. I obferved, however, that the external furface of the tumour was not fo irregular as fcirrhous tefticles generally are; but its extreme hardnefs and other fymptoms peculiar to fcirrhus were fufficiently well marked to juftify the operation.

On the 5th of June, Mr. Chambers wrote me, that he had extirpated the teftis on the preceding day. He alfo did me the favour to fend above half the amputated part, the other half having been preferved by the patient himfelf. I had the pleafure to find that the moft interefting mafs of the difeafe was included in the portion I received: for the greater part of its fubftance, efpecially the fuperior part, was granulated and femi-cartilaginous in its texture, as if interfected with offeous filaments of a light brown appearance; but in the centre of the inferior portion was a livid, pulpy, and irregularly fibrous mafs, nearly of the fize of a walnut, almoft furrounded by tranfparent fpherical veficular bodies, moft of which were not larger than fmall peas, perfectly detached from the adjacent fubftance, and from each other, and, when punctured, difcharging a pellucid fluid with confiderable force.

I ftate the appearances as I found them, and, upon the whole, cannot but imagine the above cafe to be one of thofe which Dr. Adams would adduce in fupport of his fentiments. Of this, however, I leave you to judge. I certainly believe it was a genuine carcinomatous affection, and that the cyfts were fuch as many perfons would denominate hydatids.

I remain, &c.

W. BLAIR.

*Great Ruffel Street, Bloomfbury,*

*July* 20, 1801.

